# Supplementary material for: Developed meloxicam loaded microparticles for colon targeted delivery: Statistical optimization, physicochemical characterization, and in-vivo toxicity study
Source: PLoS One. 2022 Apr 25;17(4):e0267306. doi: 10.1371/journal.pone.0267306 (PMC9037944; doi:10.1371/journal.pone.0267306)
Supplement: Attachment — Submitted filename: Responses to Reviewers.docx [file pone.0267306.s002.docx]

**The Editor-in-Chief**

**Journal PLOS ONE.**

**Subject: Revision of manuscript, (submission ID: [PONE-D-21-36281] R1)**

We are glad to submit the revised manuscript entitle “Developed meloxicam loaded microparticles for colon targeted delivery: statistical optimization, physicochemical characterization, and in-vivo toxicity study”, submission ID: [PONE-D-21-36281] R1, for publication in your esteemed journal. We are thankful to reviewers for their valuable time and useful contribution, as their inputs have helped us to improve the manuscript. We have revised the manuscript as advised by the reviewers and added the pointwise response to all the comments. Corrections made in the revised manuscript are kept as highlighted.

**Reviewers' comments:**

**Reviewer 1**: Dear Authors, this is an interesting study and the authors have collected a unique dataset using cutting edge methodology. The paper is generally well structured. However, in my opinion the paper has some shortcomings in regards to some text, and I feel this dataset has not been utilized to its full extent. Below I have provided numerous remarks on the text.

**Author response and action taken**: Thanks to the valuable feedback and comments of the worthy reviewer, the corrections have been made and the manuscript has been revised and updated thoroughly as suggested by the reviewer.

1- In line (1), Let the title tells what you did on Meloxicam loaded microparticles. I suggest “Developed meloxicam loaded microparticles for colon targeted delivery: statistical optimization, physicochemical characterization, and in-vivo toxicity study”.

**Author response and action taken**: Title has been modified in line 1 at page 1, as suggested by the reviewer.

2- The complete name of the HPMC should be written in the first time you stated.

**Author response and action taken**: The complete name of the HPMC has been written in line 13-14 at page 1, as suggested by the reviewer.

3- In line (27), replace “predicted” to “confirmed”.

**Author response and action taken**: word predicted has been replaced by confirmed in line 27 at page 1, as suggested by the reviewer.

4- In the introduction sector, what is the problem which the study try to solve or what is the objective of the work.

**Author response and action taken**: The proposed detail has been added in the introduction section, in line 71-79 and 120-130 at page 3, as suggested by the reviewer

5- In the introduction sector, authors should include literature review on the topic of the manuscript. microparticles and HPMC microparticles.

**Author response and action taken**: literature review on the topic of manuscript, microparticles and HPMC microparticles has been added in line 87-94 at page 3 & 4 and 110-111 at page 4, as suggested by the reviewer.

6- In materials and methods sector, what is the base you depend on selecting the value of the independent variables.

**Author response and action taken**: Recommended explanation has been added in line 149-150 at page 6 as suggested by the reviewer.

7- The included figures (figures 5, 6, 7, 9, and 10) are not clear). Authors should add images for suitable resolution.

**Author response and action taken**: Clear and high-resolution images have been added (now, figure 1,2,3,5 and 10), as suggested by the reviewer.

8- The magnification power of figure 4 should be included and clear for all the microparticles images.

**Author response and action taken**: The clear images have added (now figure 9), as suggested by the reviewer.

9- Figure 4 indicates that there is microparticles agglomeration.

**Author response and action taken**: Agglomerated microparticles figure has been removed.

10- In line (56), replace “drug” with “MLX” in the equation used for the Entrapment Efficiency determination.

**Author response and action taken**: The word drug has been replaced with MLX in line 201 at page 9 as suggested by the reviewer.

11- In lines (110, 111), is this (1) related to references or the number of the equation. I guess it is relation the equation so it should be differentiated from the format used with references.

**Author response and action taken**: References style was changed as per journal guideline, so now it will be differentiated from equation numbers, as suggested by the reviewer.

12- In lines 111, 140, and 150, 167 207 the same notice as before.

**Author response and action taken:** References style was changed as per journal guideline, so now it will be differentiated from equation numbers, as suggested by the reviewer, as above.

13- In line (194), write the complete letters of the word “eq”.

**Author response and action taken:** The complete letter equivalent has been written in line 245 at page 10, as suggested by the reviewer.

14- In line (220), write little details about the gastric gavage, including needle used.

**Author response and action taken:** The required details have been added in line 290-291 at page 12**,** as suggested by the reviewer.

15- In line (235), write “the optimized MLX loaded microparticles” instead of “microparticles” alone.

**Author response and action taken:** The optimized MLX loaded microparticles have been written in line 289-290 at page 12, as suggested by the reviewer.

16- In line 244, In the results and discussion section, write some introductory sentence including the aim of the study and that you prepared the microparticles.

**Author response and action taken:** Introductory sentence has been added in line 210-316 at page 13 as suggested by the reviewer.

17- The discussion lakes relation with the literature review and more mechanistic discussion is required.

**Author response and action taken:** Discussion has been made mechanistic all over discussion section, as suggested by the reviewer.

18- In lines (449 and 450), include the name of the obtained microparticles and the name of the animal used in the experiment.

**Author response and action taken:** The names have been added as in line 575-576 at page 25, **as** suggested by the reviewer.

19- Generally, more clarification is required in terms of the language.

**Author response and action taken:** The clarification regarding language has been made as suggested by the reviewer

**Reviewer 2**

In this study authors developed meloxicam (MLX) loaded HPMC microparticles for colon targeted delivery to treat colorectal cancer (CRC). The physicochemical characterizations of the developed formulations were conducted using suitable methods and the toxicity study was performed using an animal model. This work is well-designed but it has significant lacking in methodology, data presentation and overall writing. Although different organs have been examined to check the toxicity/biocompatibility of the formulation, authors did not conduct the study to see how much drug is going to the colorectal area which is the main target for this development. The manuscript is poorly written and the “Results and discussion” section doesn’t have the flow/coherency. Moreover novelty/importance of this study has not been clearly mentioned in the manuscript. Following issues need to be addressed.

**Author response and action taken**: Thanks for highlighting the main corrections. The authors appreciate the valuable feedback and suggestions of the worthy reviewer. As per the recommendation of the reviewer, all the corrections have been done throughout the manuscript.

**General comments:**

-English needs to be improved.

-Typos need to be corrected

-Inconsistencies in using terminologies (mentioned in specific comments), references need to be addressed.

**Specific comments:**

Abstract

Page 1, line 12, Need to add e after “Th”

**Author response and action taken:** Typographical mistake has been removed in line 13 at page 1, as suggested by the reviewer.

Page 1, line 21-Need a full stop after performed

**Author response and action taken:** Full stop has been added in line 22 at page 1, as suggested by the reviewer.

Page 1, lines 23-25, since the results were mentioned as range, don’t need the standard deviations and start from low to high values. e.g. the percentage yield is 65.75-90.71% etc.

**Author response and action taken:** Changes have been made in line 24-26 at page 1, as suggested by the reviewer**.**

**Introduction**

In the first para need to mention what are the current treatments for CRC then how MLX is related to the CRC treatment.

**Author response and action taken:** Treatments have been added in line 49-62 at page 2 & 3, as suggested by the reviewer**.**

In the 2nd para mention, everything about MLX. E.g. chemistry, what study (I mean formulation work) has been done on MLX related to CRC treatment, limitations of the existing research on MLX etc.

**Author response and action taken:** Changes have been incorporated in line 66-69 and 71-79 at page 3, as suggested by the reviewer**.**

Currently the importance of this work is not clearly mentioned, so bring that discussion in the introduction to show the importance/novelty of this work.

**Author response and action taken:** A discussion have been incorporated in line 80-94 at page 3 & 4 as suggested by the reviewer**.**

Page 3, line 59, what do you mean by controlled liberation, is it controlled release? If so, mention controlled release otherwise it is confusing the readers. Throughout the manuscript mention-controlled release.

**Author response and action taken:** Controlled release has been mentioned in all manuscripts as suggested by the reviewer**.**

Page 3, line 78, I think the word should be statistical not statical

**Author response and action taken:** Typographical mistake has been removed in line 112 at page 4, as suggested by the reviewer.

Page 3, lines 80-81, this is an incomplete sentence. Mention targeting of what…

**Author response and action taken:** For more clarification, a new paragraph has been added in line 120-130 at page 5.

**Materials:**

Please keep consistency in mentioning the manufacturer’s name. Currently for some you mentioned city and country and others are only country.

**Author response and action taken:** Changes have been made in line 134 and 140 at page 5 as suggested by the reviewer.

Page 4, line 91, need a comma before Quetta

**Author response and action taken:** Comma has been added in line 135 at page 5, as suggested by the reviewer

Page 4, line 93, (England, UK), is England a city here?

**Author response and action taken:** Correction has been made in line 137 and 139 at page 5**,** as suggested by the reviewer.

Page 4, line 94, delete comma before (Germany)

**Author response and action taken:** Comma has been deleted in line 138 at page 5, as suggested by the reviewer

**Method:**

Page 4, lines 99-100, this sentence doesn’t make any sense. Please rewrite this. In addition, in this sentence it should be statistical tool not statical tool

**Author response and action taken:** Changes have been made in line 146-147 at page 6, as suggested by the reviewer.

Page 4, lines 100-101, If I don’t do any mistake, for 3-level and 3-factor design total number of formulations should be 27. Why only seventeen formulations were prepared?

**Author response and action taken:** In the BBD 5 is taken as the center point, so with 5 center point total formulations become 17, because the number of formulations depends upon the number of the center point in the design.

Please keep consistency in mentioning the terminologies, either use variables or parameters but not mixed-up of both which is confusing.

**Author response and action taken:** Recommendations have been complied in all manuscript, as suggested by the reviewer.

Page 4, lines 108-109, “….practicing State-Ease…” Please clearly mention in the sentence what is State-Ease?

**Author response and action taken:** Details have been incorporated in line 157 at page 5, as suggested by the reviewer.

Page 5, Table 1, the unit for Mean particle size should be µm not µg.

**Author response and action taken:** The unite (µm) have been added in table 1, as suggested by the reviewer.

Page 7, line 159, please mention what type of particle size was measured, like volumetric, geometric etc.

**Author response and action taken:** Type has been mentioned in line 210 at page 9, as suggested by the reviewer.

Page 8, section 2.3.5, need to re-write this section in a good English. Some information are repetitive and some sentences are incorrect. How the optimized formulation (F0) came here? There is no information about F0 in the Table 1.

**Author response and action taken:** The section 2.3.5 has rewritten in line 223-227 at page 10**,** and information about F0 was given in table 1**,** was as suggested by the reviewer.

Page 8, section 2.3.6, please add the information about scanning speed and step for XRD runs. It is not clear what did you mean by measured amount, please mention approximate amount (e.g. 5-10 mg). Need to re-write this section in a good and correct English.

**Author response and action taken:** The section 2.3.6 has rewritten in line 229-233 at page 10 with required information**,** as suggested by the reviewer.

.

Page 8, section 2.3.7, please mention the different resolutions/magnifications used to collect the images. What did you mean by optimized quantity, did you try different amount of powder to make SEM samples and found out the optimized quantity? If not just mention powders samples were mounted……Also “….the stub was covered with gold using SEM” is not correct. I imagine the stub was coated with gold using as specific coater, please mention correctly with the name of the coater.

**Author response and action taken:** The section 2.3.7 has rewritten in line 235-239 at page 10 with required information**,** as suggested by the reviewer.

Page 9, section 2.3.8, It is not clear why in different phases, different mediums were used for different times. Line 205, what is configuration curve, did you mean calibration curve, if so mention calibration curve. Again delete drug liberation, instead use drug release throughout the manuscript.

**Author response and action taken:** As three different types of buffers were used for 24hrs, but phases were added for some elucidation, now it has been removed because it causing ambiguity. The other changes have also been made in line 259 at page 11, as suggested by the reviewer.

Page 9, section 4-The numbering of this section and following sections is wrong. Please correct it.

**Author response and action taken:** The numbering has been corrected as suggested by the reviewer.

**Results and discussion:**

Page 10, line 255-correct the word peek to peak

**Author response and action taken:** Typographical mistake has been removed in line 500 at page 22, as suggested by the reviewer.

Page 11, line 260, No prior information mentioned, how the optimized formulation was obtained. What happened to all other formulations mentioned in Table 1?

**Author response and action taken:** Information has been added that the optimized formulation was obtained by design from out of all (17) formulations mentioned in Table 1, in line 122 at page 5.

Page 11, line 260-261, this statement may not be correct. Why some peaks are absent in F0 which were present in the physical mixture and even in the MLX and HPMC, need explanation. Does it mean interaction?

**Author response and action taken:** The said peaks in F0 are present but inadvertently could not be labeled, now it has been labeled in fig 6, and explained in line 506-509 at page 22.

Page 11, lines 271-275, The reduction in the intensity of some peaks could be due to the difference in the ratio of MLX and HPLC in the physical mixture and F0 than the only MLX and HPMC. However, there is an absence of the first peak (at around 5 2theta position) in the F0 which is present in physical mixture as well as MLX diffractograms. Does it mean interaction? Please discuss correctly.

**Author response and action taken:** Changes have been made and discussed in line 524-529 at page 23, as suggested by the reviewer.

Page 12, lines 286-291, Again, both in the physical mixture and F0, there is a sign of interaction? Please justify this findings with other solid-state characterizations (XRD and FTIR).

**Author response and action taken:** Changes have been made and explanation has given in line 536-546 at page 23, as suggested by the reviewer.

Page 12, lines 295-296, “…..mainly depends on the polymer concentration and stirring speed of the system” How do we know that since no data on different speed and concentration has been shown here. Why suddenly F1 came in this study while for XRD, FTIR, and DSC, only F0 has been shown.

**Author response and action taken:** It was commonly assumed from the SEM images that the high polymer conc and speed images are better than low, which is also confirmed by earlier reported studies. The F1 image has been changed with another F0 image to remove inconsistency, in fig 9, section 4.6 (SEM analysis), at page 24.

Page 12, lines 302, the range should be mentioned from low to high, e.g. the percentage yield (R1) ranged from 65.75-91.71%%. Figures 5-7 and 9 are not clear. Please replace with high resolution and clear images.

**Author response and action taken:** Changes have been made in line 312 at page 13, clear and high-resolution figure (now fig 1,2,3 and 5) have added, as suggested by the reviewer**.**

Page 19, Table 5, delete the units from the column “Observed responses” as units are already mentioned in the first column “Dependent variables”

**Author response and action taken:** Changes have been made in table 5, as suggested by the reviewer.

Pages 12 to 19- From “Box Behnken Design to “Optimization of formulation” should be moved before all other solid-state characterizations, I mean before FTIR, XRD, DSC etc.

**Author response and action taken:** Changes have been made at page 13-21, as suggested by the reviewer.

Table 6, please mention the unit (e.g. mg/g etc.) for the “Weight of Rabbit Organs”

**Author response and action taken:** unite has been mentioned in table 6, part-III, as suggested by the reviewer.

**Conclusion:**

Page 21, lines 457-458, “Physicochemical characterization of formulations by FTIR, XRD, and DSC analysis showed that all components of the formulation are compatible”-this is not correct, since at least in DSC and FTIR there is an evidence of interaction. Please state whatever is in the data, don’t overstate.

**Author response and action taken:** The ambiguities have been removed in the concerned section, as suggested by the reviewer.

**References:**

Inconsistency, some has journal names and some are missing. Please keep it consistent according to journal guidelines.

**Author response and action taken:** journal names have been added in all references, and other change were also made according to journal guideline from page 28-31, as suggested by reviewer.

Thank you for your kind cooperation. We look forward to receive a good response from you.

With best personal regards.

Yours sincerely,

***Correspondence:**

Dr. Syed Umer Jan

Professor

Ph.D. (Pharmaceutical Sciences)

Post Doc (Pharm. Nanotechnology)

Department of Pharmaceutics, Faculty of Pharmacy and Health Sciences, University of Balochistan, Sariab Road Quetta, Pakistan.

Cell: +92-300-9382344

Email: [umer.pharm@um.uob.edu.pk](mailto:umer.pharm@um.uob.edu.pk)
